# Achieving Spread, Scale Up and Sustainability of Video Consulting Services During the COVID-19 Pandemic? Findings From a Comparative Case Study of Policy Implementation in England, Wales, Scotland and Northern Ireland

**DOI:** 10.3389/fdgth.2021.754319

**Published:** 2021-12-20

**Authors:** Sara E. Shaw, Gemma Hughes, Joseph Wherton, Lucy Moore, Rebecca Rosen, Chrysanthi Papoutsi, Alex Rushforth, Joanne Morris, Gary W. Wood, Stuart Faulkner, Trisha Greenhalgh

**Affiliations:** ^1^Nuffield Department of Primary Care Health Sciences, University of Oxford, Oxford, United Kingdom; ^2^Policy Team, Nuffield Trust, London, United Kingdom; ^3^Joint Research Management Office, Barts Health NHS Trust, London, United Kingdom; ^4^Independent Research Consultant, Birmingham, United Kingdom

**Keywords:** video consultations, spread, national policy, infrastructure, comparative national analysis, UK, crisis management, implementation

## Abstract

Requirements for physical distancing as a result of COVID-19 and the need to reduce the risk of infection prompted policy supporting rapid roll out of video consulting across the four nations of the UK—England, Northern Ireland, Scotland and Wales. Drawing on three studies of the accelerated implementation and uptake of video consulting across the four nations, we present a comparative and interpretive policy analysis of the spread and scale-up of video consulting during the pandemic. Data include interviews with 59 national level stakeholders, 55 health and social care staff and 30 patients, 20 national documents, responses to a UK-wide survey of NHS staff and analysis of routine activity data. Sampling ensured variations in geography, clinical context and adoption progress across the combined dataset. Comparative analysis was guided by theory on policy implementation and crisis management. The pandemic provided a “burning platform” prompting UK-wide policy supporting the use of video consulting in health care as a critical means of managing the risk of infection and a standard mode of provision. This policy push facilitated interest in video consulting across the UK. There was, however, marked variation in how this was put into practice across the four nations. Pre-existing infrastructure, policies and incentives for video consulting in Scotland, combined with a collaborative system-level approach, a program dedicated to developing video-based services and resourcing and supporting staff to deliver them enabled widespread buy-in and rapid spread. In England, Wales and Northern Ireland, pre-existing support for digital health (e.g., hardware, incentives) and virtual care, combined with reduced regulation and “light touch” procurement managed to override some (but by no means all) cultural barriers and professional resistance to implementing digital change. In Northern Ireland and Wales, limited infrastructure muted spread. In all three countries, significant effort at system level to develop, review and run video consulting programs enabled a substantial number of providers to change their practice, albeit variably across settings. Across all four nations ongoing uncertainty, potential restructuring and tightening of regulations, along with difficulties inherent in addressing inequalities in digital access, raise questions about the longer-term sustainability of changes to-date.

## Introduction

With a view to containing novel coronavirus (COVID-19), healthcare organizations across the world rapidly introduced new service models in 2020 intended to help avoid in-person clinician-patient contact and reduce the risk of transmission. Video consulting was a key part of this major service innovation, involving rapid and widespread logistical, cultural and technical change (1–3) and redefining what an accessible and technology-enabled health service looks like (4–10). Set up of video consulting services has been widespread during the pandemic, with adoption and use varied across countries and clinical settings (11).

Pre-pandemic, adoption of video consulting was slow, time-consuming and resource intensive, with activity limited to specific clinical services and settings (typically with a local clinical enthusiast leading). Evidence on the use of video consultations in health services was mixed (1, 12–14). There was a small but rapidly growing literature on feasibility and acceptability of video consultations across clinical areas [e.g., diabetes (15, 16), ophthalmology (17), cancer (18, 19) and therapies (20–22)]. Patients generally welcomed video consulting services (23–25). While evidence supported the potential of video consulting in small scale implementations, little was known about how to successfully spread, scale-up and sustain it. What little research there was tended to adopt a technology-centric approach (in which the technology is the primary focus, rather than the service or organization in which the technology is being used) and use trial methodology to study whether video consultation technology does or does not work (1). Studies were often small scale and focused on initial adoption in the context of a research study (26, 27). Video consulting services frequently encountered difficulties when attempting spread in “real world” complex health systems (28–30). There was limited formal evaluation of policy initiatives supporting spread and scale-up of video consulting, with political and policy realities and institutional structures typically sidelined or ignored (30, 31).

This has begun to change in the context of the COVID-19 pandemic, what scholars on crisis management would describe as a highly unusual and volatile situation with potentially far-reaching negative implications (32–34). Such crisis situations—“*critical junctures in the lives of systems*” [(32), p. 6]—are characterized by *threat* (in which the core values or life-sustaining systems of a community are put at risk), *uncertainty* (about the nature of the threat and/or possible consequences), and *urgency* (in terms of being here and now, and needing to be urgently managed). Response requires policymakers to rapidly mobilize multiple organizations and sectors, and align different professional logics and ways of working; grapple with a complex and multifaceted picture of proximal and distal concerns; contemplate actions that would otherwise not be on the policy radar (e.g., introducing “lockdowns”); and, guided by national leadership, act rapidly and at scale through “*field operations and in networks that lack clearly defined authority relations*” [(32), p. 17]. As we set out below, in the context of the pandemic, this led to rapid rollout of telehealth initiatives and digital services, an urgent need for health systems frameworks that could support spread, and an explosion of research on the rise of telehealth (35). The later has typically focused on demand trends, rapid set up and adoption, implementation at scale and pace, and future sustainability of services (11, 36–43). While national policy and regulation are frequently acknowledged as integral to spread and scale up (e.g., 36, 40), the everyday practices allied to policy implementation have rarely featured. Requirements are frequently set out (e.g., funding for telehealth services, training for health professionals, redesign of clinical care) but, as Smith et al. (36) so neatly put it, the assumption is that “*the consideration of whether telehealth could be used in emergencies [is] redundant as it should just happen*” (p. 4). Similarly, literature on policy and health innovation tends to focus on policy formulation or how specific discourses shape ideas about innovation [see e.g., (44, 45)]. In sum, existing evidence tells us little about how to implement policy on the rapid spread and scale up of video consulting during a time of crisis.

In this paper we unpack what could or should happen to achieve spread and scale up of video consulting services during a time of crisis. Much earlier research and analysis takes policy as a technocratic process involving a set of given steps or stages—and the transition from *policy* to *practice* as somehow given. Drawing on an interpretive approach to policy analysis (46, 47) we challenge this, seeking to unpack the black box of policy implementation during the pandemic.

Our focus is on the UK National Health Service (NHS). In the context of COVID-19, policy across the UK four nations has been to facilitate roll out, spread and scale-up of remote consulting as a means of managing the risk of infection while continuing to deliver safe and accessible health care (48). This was overseen by the then Secretary of State for Health, Matt Hancock, who took up position in 2018 with the ambition of making the NHS more digitally enabled (earning him the nickname “Matt The App”)—since the start of the pandemic in 2020 he repeatedly called for NHS services to become “remote by default” (49, 50).

Despite significant take up of video consulting across the UK since the start of the pandemic (often, but not always, building on adoption pre-pandemic), there has been variation in the speed and scale of set up and spread. This raises questions about why some countries were more able to rapidly implement video consulting when policy across the four nations supported use of video consulting as an important means of managing the risk of infection during the pandemic. We therefore ask:

1. What is the social and policy context shaping video consulting across the UK?
2. What has been the approach to enacting policy during the COVID-19 crisis, and how has this shaped, enabled and constrained the spread and scale-up of video consulting across the UK?
3. What lessons can we learn from comparative analysis of policy implementation guiding the spread and scale-up of video consulting across four UK nations, and how might this inform sustainability of services beyond the pandemic?

In the Methods section below we set out the methods used, drawing on three on-going studies focused on remote and video consulting across the UK, and detail our theoretical and methodological approach grounded in interpretive policy analysis. In the Results section, we present case studies of the spread of video consulting in each of the four nations as well as cross-national comparison. In the Discussion section, we discuss the implications of our findings for future spread, scale-up and sustainability of video consulting services and for policy implementation on video consulting.

## Methods

### Study Design and Use of Existing Datasets

Building on 10 years of research on video consultations [e.g., (1, 51–53)], our focus was on the extent to which the evolving crisis and policy response has shaped spread and scale-up of video consulting in England, Scotland, Wales and Northern Ireland, each being a member of the UK with varied devolved legislative powers and political processes (Table 1).

**Table 1:** Overview of the structure of health systems and selected health and healthcare indicators in each of the UK four nations^*^.

	**England**	**Scotland**	**Wales**	**Northern Ireland**
**Health system structure**				
Government department	Department of Health and Social Care	Health and Social Care Directorate	Department of Health and Social Services	Department of Health
Purchaser-provider split	Yes	No	No	Yes (in theory, but not always in practice)
Main bodies involved in commissioning and planning services	NHS England; clinical commissioning groups; local authorities; Public Health England	Seven special NHS boards^*^; Public Health Scotland; joint boards comprised of 14 regional health boards and local authorities (i.e., social care)	Three NHS Trusts; Welsh Health Specialized Services Committee; seven regional partnership boards (seven local health boards and local authorities)	Health and Social Care Board; Public Health Agency; five local commissioning groups
Main organizations with scrutinizing or regulatory roles	Care Quality Commission (i.e., all health and care services: public and private); NHS England/Improvement	Healthcare Improvement Scotland (i.e., healthcare services: public and private)	Healthcare Inspectorate Wales (i.e., all health-care services: public and private)	Regulation and Quality Improvement Agency (i.e., all health and care services: public and private)
**Financing model, expenditure on health and entitlements**				
Predominant model of financing	General taxation	General taxation	General taxation	General taxation
Spending on health per capita (financial year 2017–2018), £	2,168	2,353	2,310	2,306
Annual spend on private health insurance per household	104	36	62	47
**Workforce**				
General practitioners per 1,000 people, 2018	0.58	0.76	0.63	0.67
Hospital consultants per 1,000 people, 2018	0.88	1.04	0.86	0.96
Nurses per 1000 people, 2018	6.60	9.07	8.36	9.16
**Population and demographic characteristics, 2019**				
Population size, millions	55.98	5.44	3.14	1.88
Population density, people per km	432	70	153	137
Proportion of pop'n 65 or over, %	18.4	19.1	21.0	16.6
Proportion of pop'n 85 or over, %	2.5	2.3	2.7	2.0

* *Adapted from the LSE–Lancet Commission on the future of the NHS*.

In 1999, the UK devolution settlement created autonomous, elected governments for Northern Ireland, Scotland and Wales and transferred powers for health from the Westminster UK Parliament to the Scottish Parliament, Welsh Assembly, and Northern Ireland Assembly (54). Many challenges have since remained in common (e.g., developing and implementing effective information technology) (55). However, devolution inevitably led to further divergence in health policy (56). The ways in which each of the four nations negotiated the pandemic therefore provided a naturally-occurring opportunity for comparative analysis. To do this we drew together datasets from three separate studies, each examining video consulting in one or more parts of the UK.

First, we undertook a national evaluation of video consultation services in Scotland in early 2020, funded by the Scottish government. This work involved quantitative and qualitative data collection and the production of narrative case studies to illustrate the successes, failures and partial successes of efforts to use this technology in different settings and services. The Scottish example is interesting because much work was put into building a national infrastructure and branding for the video consultation model (48). We were then commissioned by the Scottish Government to extend this work and document how things changed during the COVID-19 response.

Second, we conducted a longitudinal analysis of the scaling up of video consultations across the UK during the pandemic. Funded by the Health Foundation, we extended the work already conducted in Scotland to include England, Wales and Northern Ireland by conducting a UK-wide survey on video consulting, along with follow up interviews with NHS staff, and interviews with patients and national-level stakeholders. This allowed us to understand the extent to which the evolving crisis shaped scale-up and gain transferable insights into the development of sustainable service models. It also provided comparisons across the four nations.

Finally, we received funding from the UK Research and Innovation emergency response fund to look at “remote by default” primary care in the context of the pandemic. Because COVID-19 is so contagious, patients could no longer walk into a GP surgery and ask to be seen but had to apply online or phone the surgery for an appointment. Our focus in this paper is on the macro (national infrastructure) aspects of a remote-by-default service model in primary care that supported these rapid and widespread changes and sought to rapidly strengthen the supporting infrastructure for digital innovation in the NHS. Other elements of the research focused on the micro- (technical tools, clinical techniques), and meso-(organizational change) aspects of remote by default consulting and supporting the change process through action research and are reported elsewhere [e.g., (57, 58)].

As detailed in another paper in this special issue (57), these three studies all addressed—in one or another version—the following question: “what are the challenges—at individual, organizational and system level—of introducing remote consultation services at pace and scale and routinizing such services to become business as usual?” Data included here were collected over a 20-month period (January 2020 to August 2021), capturing the start of the COVID-19 pandemic and on-going progress. All three studies included an explicit policy (macro-level) component.

### Theoretical and Methodological Approach

We turned to interpretive policy analysis (46, 47, 59) to guide our understanding of the policy process, particularly policy implementation (i.e., the actions and interactions that bring policy into being). Through this lens “policy” is a set of processes and actions (or inactions) that have some broad purpose rather than, say, a discrete decision or program administered at a particular moment in time, and emerges rather than being predetermined (59, 60). The health system can be thought of as a complex and dynamic network of actors, practices and interactions (61, 62), with control typically dispersed and the direction of the system shaped by multiple decisions and interactions of varied actors. This theorization is critical for our study of policy implementation. The policy process is recognized to be more complex than previously understood, with earlier literature on the “policy-implementation gap” now supplemented by complex systems thinking informed by notions of unpredictability, non-linearity and adaptability (63). Here the factors that shape and influence implementation are seen to be complex, multifaceted and multileveled (60, 64).

This focus on complexity and social action stands in stark contrast to the prevailing view of policy as a formal, rational process that can be planned in advance. This is deliberate. Scholars of health policy have often [but not always, see e.g., (65)] aligned themselves with an instrumental approach that situates individuals and institutions within a “rational choice” framework [see (66–68) for a detailed review]. What follows is a tendency to see policy as somehow separate from politics, and policymaking as a linear process involving problem identification, collection of data on alternative solutions, and selection of the alternative that best resolves the problem (46, 69). Such an approach focuses on the instrumental goals that people seek to achieve (e.g., influencing specific policies); assumes that policy actors generate “objective”, policy-relevant knowledge in a void; and tends to adopt quasi-experimental designs and quantitative methods to evaluate the goals of policy programs. In contrast, our focus is on social actions that contribute to the meaning of policy (70), the role of varied actors [from national-level political actors to “street level bureaucrats” (71)], and the interactions, values and processes involved in enacting it.

### Sampling and Data Collection

An interpretive approach recognizes policy as negotiated and renegotiated in the social practices and encounters of administrators, regulators and other street level bureaucrats (46, 59, 72) (e.g., those liaising with suppliers, rolling out software or tracking and reviewing activity). We therefore focused data collection on national-level policy and planning and organizational-level enactment. Data sources are summarized in Table 2 and described in more detail below.

**Table 2:** Overview of data sources and analysis.

**Data source**	**Data collected**	**Contribution**
UK-wide evaluation of spread and scale-up of video consulting	Accounts of 59 senior-level, national stakeholders involved in digital health and video consulting (17 in England, 12 in Wales, 7 in Scotland, 5 in N. Ireland, and 18 with UK focus), including:	Social and political context, including rapid onset and evolution of COVID-19 pandemic
	• 21 civil servants/policymakers	Policy and regulatory drivers, system-level and infrastructure blocks and changes over time
	• 15 professional groups	Logics by which spread and scale up of video consulting have been planned and put into practice
	• 12 business and industry	Reflections on longer term planning and the role of video consulting across settings
	• 8 senior executives	Extent of set up, uptake and spread, timeframes, geographical distribution and patient demographics; and any changes over time
	• 3 patient representatives	
	20 documents, outlining policy and guidance on digital health and video consulting across the four nations	
	Quantitative data and reports on activity	
Staff and patient experiences of video consulting	Responses from UK-wide survey of NHS staff (n=809) about adoption and use of video consulting, with 52% of responses from NHS staff in England, 35% from Scotland, 8% from Wales and 5% from NI.	Sense-making about the design, delivery, experience and spread of video consulting services in the context of COVID-19, including national and inter-organizational networks, policy directives and regulation
	Accounts from 40 (clinical and non-clinical) staff across the four UK nations, including:	Acceptability/popularity of video consulting services
	• 11 in Northern Ireland	Required/available human, social and financial resources
	• 9 in Wales	Changes needed to underlying infrastructures (technical, organizational, workflows)
	• 10 in England	Professional, ethical and moral questions about video consulting and rapid service change
	• 10 in Scotland	Learning shared across sites and networks
	Plus follow up interviews with 20 of these (5 in each country)	
	15 interviews with primary care staff from 8 GP practices in England involved in group video consulting	
	Accounts of 15 patients receiving individual or group consultations (or having declined the option)	
	Two focus groups with a total of 15 patients/public about engagement with, and experiences of, video consulting	

We identified national level stakeholders through a mix of purposive, snowball and maximum variation sampling. We began by connecting with teams conducting policy relevant work on technology implementation and digitally-enabled care (e.g., NHS England, NHS Scotland), inviting individuals to participate in interviews and asking for nominations of further people that they recommend we speak with. To spread the net wider, we reviewed policy documents and staff interviews (see below) for mention of individuals, teams or organizations leading work on the spread of video consulting and invited them as further interviewees. This gave a broad sample across civil service, professional and patient groups, regulators and industry. Some interviewees were able to give a UK-wide perspective (e.g., from industry), others a national perspective. For the latter, we reviewed our sample across the four nations and then actively sought interviewees who were able to fill any gaps (e.g., interviews in Northern Ireland tended to focus on secondary care, leading us to proactively identify primary care professionals leading technology-enabled change). This provided a final sample of 59 interviewees (Table 1).

We tracked evolving policy in the four nations and asked interviewees to suggest relevant documents, resulting in a sample of 20.

The survey focused on spread and scale-up of video consulting during the pandemic, aimed to capture NHS staff experience across the UK and was designed using SurveyMonkey with input from Barts Health NHS Trust (JM), NHS England, NHS Scotland, NHS Wales and NHS Northern Ireland [see (73) for link to final version]. We used a combination of opportunity and snowball sampling to distribute the survey to NHS staff across the UK, using NHS and research networks (full list available from authors). The survey was also distributed *via* social media, with targeted tweets aimed at increasing diversity of respondents (e.g., geographical areas, specific groups; e.g., LGBTQ NHS networks). The survey was live for 3 weeks in September 2020.

We asked survey respondents to indicate if they would be prepared to be contacted for interview, then selected 40 ensuring maximum variation of country, organizational and clinical setting, role (clinician, support staff or manager) and rural/urban location. Patients were recruited *via* Patient and Public Involvement (PPI) networks in London and Oxford, the NHS England public participation team and voluntary sector organizations. We sought maximum variety in terms of age, ethnicity and location and ensured representation from health advocates to capture views from people who were not able to use remote methods of interviewing.

We adopted a narrative approach to interviewing, aiming to capture the story of how video consulting developed before and during the pandemic, experiences of this and perspectives on if/how health system policies and incentives enabled spread and scale-up of video consulting within and across the four nations of the UK. We interviewed five national level stakeholders and 20 NHS staff twice to capture accounts over time. We held two online focus groups to share emerging findings and discuss views and experiences of video consulting.

### Analysis

SS led the analysis, working with a core analytical team and following an interpretive approach (46, 47, 70). This involved initial thematic analysis of qualitative data. Quantitative data were aggregated and analyzed using basic statistical methods. Guided by the “wider system” elements of the PERCS (Planning and Evaluating Remote Consultation Services) framework, all data were then brought together into an emerging narrative of each of the four UK nations focusing on the policy context (e.g., technology-enabled care, planetary health), infrastructural elements (e.g., broadband availability) and opportunities for cross-national influence and learning. The PERCS framework is an adaptation of a more generic framework for considering the complexities involved when introducing new technology (74) consisting of 8 interdependent domains (e.g., reason for consulting, clinical relationship)—development and rationale are explained in a separate paper (57).

We then undertook cross-case comparison, informed by dialogue with relevant theory and leading us to identify five key themes that helped to explain similarities and differences in the implementation of policy shaping the spread of video consulting during the pandemic. At the start of this process, we were struck by the ways in which some interviewees discussed the collective sense-making involved in the initial stages of the pandemic and in shaping the ways in which they negotiated and sought to implement emerging policy on video consulting. As we engaged further with our data it became clear that this differed within and across nations. We therefore drew on work on “making policy happen” (62, 64, 75) to examine the approach to putting policy into practice (before and during the pandemic), as well as the ways in which decision makers went about the work of supporting implementation.

This process raised questions about how the “crisis context” of the pandemic shaped policy implementation. To examine this we drew on a small (but growing) literature on crisis management (32, 33). This describes the features of crises—threat, uncertainty, urgency and collective stress—and the combination of critical tasks that need to be effectively managed including that critical decisions are made by the right people, the efforts of those responding are orchestrated and that government communicates with the public effectively (32, 76). As we explored the intersection between policy implementation and crisis management in our data, we identified two different approaches to crisis response that shaped how critical implementation tasks were—and weren't—accomplished. Firstly a traditional approach grounded in rationalism and focused on *principle-guided crisis management*, in which complexity is often negated and attempts are made to tame uncertainty by relying on longstanding principles and—often technocratic—ways of working (33). Secondly, a pragmatic approach in which the focus is on sense-making (e.g., enabling ongoing, collective reflection), decision-making across multiple networks, meaning making (involving credible and convincing interpretation and public explanation of a crisis), learning-while-doing and developing adaptive capability (e.g., with decision makers and health care staff trained to tinker with technologies and processes, and make judgements) (33, 61, 76). This later approach resonated strongly with the interpretive approach outlined above, the concept of intelligent policymaking (64) and the recognition that policy implementation takes place in complex systems (63, 74).

## Results

### Overview of Policy Approach to Video Consulting in Each of the Four Nations

Health systems across the UK have evolved differently (Table 1), shaped by historical and national contingencies (54, 77). Below we provide an overview of the varied development of video consulting in each of the four nations (Table 3), before presenting five cross-cutting themes.

**Table 3:** Overview of policy approaches to video consulting across the four nations, before and during the COVID-19 pandemic.

	**England**	**Scotland**	**Wales**	**Northern Ireland**
Pre-pandemic policy and infrastructure	Longstanding concern with new technology as a means of generating efficiencies, with impetus for innovation-driven change in health care, including *video and e-consulting*; early adoption of platforms in some settings; evolving but limited infrastructure	Longstanding policy vision and support for *technology-enabled care* and allied infrastructure, including Near Me, national video consulting service; significant impetus from cross-government agenda to reduce carbon emissions	Policy push for technology-enabled care, including *video consulting*; with support for local pilots, regional spread then national roll out, but limited/varied infrastructure	Policy supporting *virtual* consulting largely oriented to phone consulting; ambition for digital health, with video consulting evolving via small quality improvement programs; digital infrastructure limited with widespread absence of broadband
How the immediate crisis response was framed in relation to digital technology	*An opportunity to innovate*—to accelerate set up and spread of novel forms of remote consulting across the NHS, thereby achieving the policy goal of “remote by default”	*An opportunity to scale-up*—building on established infrastructure, to extend and learn from existing models of technology-enabled care, bringing all parts of the country to the level of exemplar sites	*An opportunity to become known as a national digital innovator*—to build national video consulting service and gain political and health system currency	*A window on challenges*—revealing gaps in infrastructure and digital readiness, as well as dilemmas about how to organize and deliver care at time of crisis
Policy and regulatory shifts during the pandemic	Centralized procurement, slackening regulation, relaxed information governance; fast-track research into remote consulting	Centralized procurement, slackening regulation, relaxed information governance; rapid evaluation and learning	Centralized procurement, slackening regulation, relaxed information governance	Slackening regulation, relaxed information governance, rapid quality improvement set up
Approach to technology supply during the pandemic	Mixed approach, with central contract to single supplier (Attend Anywhere) for secondary care, combined with encouraging other suppliers in to the wider NHS who met minimal standards and could deliver a usable product at speed	Extension of existing contract to single supplier of video consulting platform (Attend Anywhere) in strongly-branded national program (Near Me)	Mixed approach, seeking to learn from, and emulate, Scotland's success with a single national supplier while also recognizing multiple suppliers	Continued arrangements with existing multiple suppliers, with interest in learning from Scotland's success with a single national supplier
Approach to spread and scale up of video consulting during the pandemic	Rapid roll-out and implementation of innovative technologies, central support and guidance, varied procurement (e.g., locally driven in primary care, centrally steered in secondary care)	Extension of successful models of good practice using principles of quality improvement—with facilitated adoption, central support, training and guidance, and system learning	Rapid roll-out and implementation, central support and guidance, central procurement	Continued emphasis on virtual consulting with extended use of existing video platforms supported via evolving quality improvement program
Key sources of learning for national roll-out	Cross-national peers (esp. Near Me in Scotland), on-going research and evaluation, NHS data and provider feedback, industry/tech suppliers	Dedicated quality improvement cycle, involving collaboration among service leaders, capturing data in a “learning health system” model and external evaluation; sharing learning with cross-national peers	Cross-national peers (esp. Near Me service in Scotland), in-house evaluation, provider feedback	Predominantly in-house quality improvement and provider feedback, plus external input from peers in other nations (esp Near Me service in Scotland)
Adoption and use of video consulting	Wide variation by setting and specialty. Very little sustained uptake in primary care	Substantial national adoption overall, though used significantly less in primary care	Wide variation by setting and specialty. Very little sustained uptake in primary care	Wide variation by setting and specialty. Limited uptake in primary care
Longer term policy focus	Promote innovation-driven new service models, support supplier diversity, address digital exclusion, generate patient-led demand and extend video consulting services	Routinize Near Me service, ensure solid infrastructure, support patients and professionals, address health/digital inequality, evaluate and share learning; achieve carbon reduction goals	Extend national video consulting service, address digital exclusion, develop and support infrastructure	Refine and implement policy on digital health, develop digital infrastructure including strengthening broadband coverage, grow quality improvement collaborative on video consulting

#### England

England has a population of over 55 million and a large health system (Table 1), with over 200 NHS Trusts and Foundation Trusts and over 6,500 general practices. Geography is varied, including dense urban areas and remote and rural communities. There is a mixed economy of care with, for instance, multiple providers supporting services to a diverse population (>9 million) across Greater London, through to single providers supporting expansive rural areas. Video consulting technology has been available in healthcare for many years, though use has varied across specialties and settings.

While there has been overarching national guidance on remote and online consulting, there has been no defined national policy on video consulting *per se*. Since 2010 a series of announcements has emphasized digital innovation and remote care (78–83), reflecting concerns to generate efficiencies *via* use of technology, increase access and reduce the NHS's carbon footprint. In 2016 the General Practice Forward View set out plans to offer every practice support to adopt online consultation systems, committing an estimated £45 million investment (78). In 2019 the NHS Long Term Plan (79) set out the aim for up to a third of face-to-face appointments in outpatient care to be avoided by embracing technology and arranging services around patients' lives. The vision was for “digital first” primary care to become a reality by 2024.

Pre-pandemic video consulting remained a largely *ad hoc*, bottom up activity, led by enthusiasts. While there was early adoption in some settings [e.g., “Skype clinics” for young adult diabetic patients (51, 84)], limited infrastructure and the challenges of embedding video consulting in existing “in person” clinical pathways, meant slow spread to other services. The use of video consulting therefore remained relatively low until March 2020.

As the pandemic hit, NHS England and Improvement (NHSE/I, Table 1) focused on accelerating access and uptake of remote consulting, including video consulting, across the English NHS. In primary care, the NHSE/I Digital First Primary Care team worked closely with NHS Digital to set up a new, rapid procurement route for online consultation and video consultation platforms (part of the Digital Care Services Catalog), allowing commissioners to sidestep the diverse and complex supplier market and instead procure one of 11 nationally assured products. All practices were asked to rapidly shift to “total triage” (85), requiring patients to contact the practice (typically online), provide information and be triaged before making an appointment. Central guidance, combined with multi-agency regional support, aimed to facilitate implementation and service improvement, along with support from NHSX (the body supporting digital transformation in the English NHS). A separate strand of work involved commissioned training for video group clinics in general practice—a relatively new service innovation involving two or more patients and one or more clinicians.

**Figure 1:**
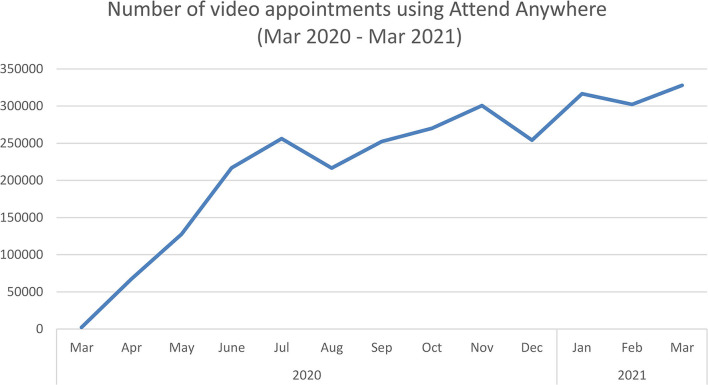
Growth of video consultations during the pandemic. Graph shows total number of video consultations for NHS hospitals in England using the Attend Anywhere platform, March 2020 to March 2021.

In secondary care the NHSE/I Technology Implementation Team had closely followed developments in Scotland (where a national video consulting service had already been established—see below), and set up several pilot video consulting services following a similar approach with the same platform. Off the back of the pilot, and under pressure to rapidly accelerate rollout to all NHS hospitals in England in 4 weeks, NHSE/I procured and funded a national license for Attend Anywhere, giving hospitals the option to use the platform for 12 months. Training and materials to support swift deployment were quickly made available, along with £20,000 funding per provider to support implementation (regardless of whether they used the Attend Anywhere platform or not), a national helpdesk, provision of over 5,000 iPads to frontline staff and negotiation of zero-rated 4G on major networks to support patient access to video services (86). The use of video consulting increased significantly, with close to 3 million video consultations *via* Attend Anywhere in 2020/21 (Figure 1). The greatest increase in activity occurred in the first month (which saw a 32-fold increase, 3130%). Growth slowed as physical distancing requirements slackened but continued steadily (Figure 1). Close to half (48%) of video consultations took place in psychology/mental health, physiotherapy and pediatric or child/young adult services. A further 1.5 million video consultations took place in the same period *via* other platforms (87).

National procurement of Attend Anywhere ended on 31 March 2021, with NHS hospitals then procuring their preferred platform (frequently Attend Anywhere), supported by central guidance and funding to 31 March 2022 (when they will need to be locally procured and funded).

In sum, remote consulting activity pre-pandemic provided foundations for rapid set up of video consulting in the English NHS. Evolving national infrastructure and a diverse supplier market proved challenging. Adoption and use varied by sector, with use growing significantly in secondary care and primary care largely declining due to use of telephone and asynchronous e-consultations.

#### Scotland

Scotland (population 5.5 million, with low density—see Table 1) has a rugged geography and outlying islands, resulting in access challenges for many people. The Scottish NHS is underpinned by a strong public-sector ethos that emphasizes professionally-led quality improvement and reducing inequalities (88). Health and care services are mainly delivered by 14 territorial health boards with remote care framed as a means to progress access to services, improve outcomes and reduce inequalities. Video consulting has long been advocated, initially *via* the eHealth Strategy in 2008 and, more recently, *via* the 2018 Digital Health and Care Strategy (89).

In 2014, the Scottish Government established the Technology Enabled Care (TEC) program, focused on driving widespread adoption of technology to support self-management of illness (e.g., self-monitoring of long-term conditions) and improve access to care. The initiative was, at least in part, a response to rising demand for health and social care and the need for service transformation. Funded by the central government, the TEC program included a series of work streams aimed at supporting local deployment, strengthening national infrastructure, and placing Scotland at the forefront of delivering technology enabled care.

The video consulting work stream was seen as enabling pooling of expertise and provision across the country to ensure high-quality patient experience. In the early years of the program, this involved various pilot studies that used varied technologies (e.g., Cisco Jabber), before the national decision was made by the TEC team in 2015 to introduce a bespoke product. Based on the success of an initial co-design and quality improvement program in one health board in 2017, a national video consulting service using the Attend Anywhere platform was then established, branded as “Near Me”. In 2018 the TEC program launched a £1.6 million ($2.3 million) “scale-up challenge” to support rollout across all health boards. By 2019, a national program to extend the service was well under way, driven by an ethos of collaborative quality improvement, reducing inequalities and achieving cross-government low-carbon goals.

Before the pandemic (i.e., by February 2020) all 14 health boards and the Golden Jubilee National Hospital (one of the main tertiary referral centers) were enrolled in the program. The Near Me video service had been adopted by about 180 services, spanning 35 different clinical and social care specialties. Levels of implementation varied: use of video within most services remained relatively low, with use largely “*ad hoc*” rather than business-as-usual. Two of Scotland's 14 territorial health boards (where enthusiasts were based) accounted for most activity, with on-going spread elsewhere supported by a national team who steadily worked through regulatory, infrastructural and operational challenges.

**Figure 2:**
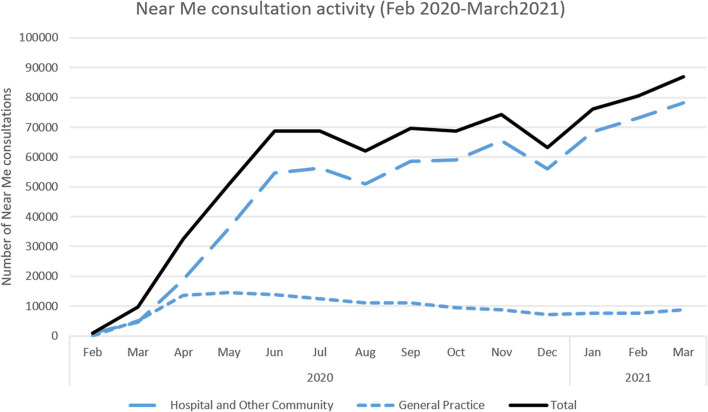
Growth of video consultations before and during the pandemic. Graph shows total number of video consultations for GP, hospital and other community services, February 2020 to March 2021.

The Scottish Government's Programme for Government 2019–2020 referred to a planned expansion of Near Me and committed to using it as a means of opening up services to those who may struggle to travel (90). When the COVID-19 outbreak reached Scotland in March 2020, this planned expansion was accelerated *via* a 12-week scale-up plan, led by a rapidly-assembled national implementation team within the TEC program. Staff were drafted in from across Healthcare Improvement Scotland (a Special NHS Board, with a remit to help implement healthcare priorities), Scottish Access Collaborative (a government program to sustainably improve waiting times for non-emergency procedures) and the Care Inspectorate (a regulatory body focused on social care services). They prepared guidance and resources for deployment of video consultations across health and care settings and built links with the government's Primary Care Division (that then mobilized resources for implementing the service in general practices), and other government departments. This led to a rapid and dramatic expansion of the service (Figure 2). Between March and June 2020, the number of video appointments increased 50-fold, from about 330 to 17,000 appointments per week nationally, with over 50 clinical specialties, across the 14 health boards, introducing video consultations for the first time. As in England, the majority of activity fell within psychology/mental health, physiotherapy and pediatric or child/young adult services.

In sum, national-level groundwork and strategic planning to create technical infrastructure, service readiness and positive attitudes to a national video service, combined with targeted implementation support, all helped services transform, at scale and at a massively accelerated pace, as the pandemic took hold.

#### Wales

Wales is a small country with a relatively dispersed population of just over 3 million, with many living in rural areas and a slightly higher proportion of older people than other nations (Table 1). There is a strong public sector ethos. Like Scotland, successive Welsh governments have elected not to follow the market-based approach of English health system reforms, focusing on co-operation rather than competition in health care. Health inequalities, and more recently digital inequalities (91), have been a longstanding concern.

Use of IT has long been on the agenda for NHS Wales (92), with technology-enabled care and video consulting part of strategy since 2015 (93). The broad aim was to use technology to “modernize” the NHS, with a focus on “implementing the technology”. A cross-party Parliamentary Review of Health and Social Care in 2018 (94), was quickly followed by publication of Healthier Wales in the same year (95), setting out government plans for transformation of health and social care. The later placed “digital and data” as central to that agenda, while recognizing significant limitations posed by existing digital and infrastructural arrangements. This provided foundations to support development of an NHS Wales Video Consulting Service, with significant work required to “*better leverage… technology and infrastructure assets*” [(95), p. 27]. In this pre-pandemic phase, bureaucracy frequently stifled innovation (e.g., with long timelines for business case development, and digital procurement), a program of work to install Microsoft Office 365 was planned but not yet actioned, and support for implementation and scale-up of digital innovations *via* the National Welsh Information Service (NWIS, the national organization responsible for building and designing digital services, now Digital Health and Care Wales) was patchy.

A new national program for Technology Enabled Care quickly followed, with TEC Cymru (a hospital-based team focused on developing and supporting technology enabled care) given a national mandate to roll-out video consulting in 2019, and central government funds available to support NHS organizations to purchase equipment. TEC Cymru's development of video consulting services was modeled on a well-trodden digital innovation blueprint in Wales involving local pilot initiatives, regional spread and national roll-out. Looking to Scotland's Near Me Service as a beacon site (48), Attend Anywhere was adopted as the platform of choice. Pilot services were set up in secondary care and community services (e.g., speech and language therapy) with a focus on supporting early adopters and generating learning. Results were promising. However, when the pandemic hit in March 2020, video consulting remained the preserve of a small group of (largely secondary care) enthusiasts.

As part of the emergency response to the pandemic the Welsh Government invited TEC Cymru to lead an accelerated national roll-out of video consulting services, across health (and social) care; initially focusing on primary and then secondary care. NHS Wales, with support from NWIS, quickly switched the Welsh NHS to Microsoft Teams (via rapid acceleration of the planned program). A government announcement of £50 million recurring funding *via* a new Digital Priorities Investment Fund a year earlier (96), in 2019, meant updates had been done to legacy hardware and software, providing a significant boost to underlying infrastructure and easing rollout. A newly formed “Digital Cell”, bringing together the central health and social care team, with digital leads from 11 organizations, met frequently each week to enable rapid decision-making.

TEC Cymru provided implementation support to providers taking up the offer to rapidly develop video consultation services, especially in secondary care. Limitations in server capacity during the early emergency period (Attend Anywhere was overwhelmed with demand), combined with kickback from the GP community who felt that Attend Anywhere was not a good “fit” with general practice (preferring other platforms that, e.g., allowed greater use of text-based and asynchronous communication), meant that the roll-out was not limited to one platform, with others (e.g., AccuRx) also in use. Interest in group video consulting grew, with training sessions commissioned to support development and rollout.

There is limited detailed evidence on how policy played out on the ground, since national level activity data is hard to come by. Data from the TEC Cymru team (who conducted an in-house evaluation of the evolving national video consulting service), reported over 38,000 video consultations using the Attend Anywhere platform across primary, secondary and community providers from the start of the pandemic to the end of August 2020 (97). To date two evaluation reports provide extensive data and useful insights into the use of Attend Anywhere during the pandemic (97, 98). However they do not report data on the use of other platforms. These documents describe many successful aspects of the program but pay scant attention to the kinds of challenges and conflicts that characterize adoption and spread of digital technology generally and that were documented across the other three nations.

In sum, the Welsh approach to developing video consulting services seems to have been characterized by tensions between those committed to delivering a national service by driving through Attend Anywhere as the main platform (as Scotland did) and others who favored a more pluralist and flexible approach to technology providers. Digital infrastructure was historically weak but had been quickly updated, allowing rapid roll-out and spread of video consulting, with coordinated national scale-up remaining a longer-term strategic objective.

#### Northern Ireland

Northern Ireland is a small country, with a population of close to 2 million around a third of whom live in rural areas. It has a rich, if complex, history. Established in 1921 when Ireland was partitioned, Northern Ireland has a turbulent, and at times violent, political history characterized by competing perspectives (mostly drawn along religious lines) on the future of Northern Ireland. The “Good Friday” agreement enabled a coalition government to be established in 1999, though this has since been suspended several times. Relationships between Northern Ireland, the rest of Ireland, and the rest of the UK are complex. Recent Brexit negotiations, which frequently placed border arrangements with the UK and Europe center stage, have not helped. Against this challenging backdrop the health system in Northern Ireland is perhaps the least developed of the four nations.

Policy on digital technology and innovation is relatively new to Northern Ireland. Pre-pandemic there was a focus on addressing demographic changes, rising demand and significant health inequalities (99, 100). Proposals for service transformation recognized the value of innovation and the need to maximize use of technology (100, 101). However, while there was an ambition—and growing political support—for digital health; limited capacity, restricted resources and a lack of nationally-coordinated digital infrastructure meant a disconnect between high level policy and frontline practice. At this time the focus was on virtual consulting, which largely (though not completely) equated to telephone consulting (e.g., “*when we talk about virtual consulting in Northern Ireland, we're talking about telephone or video*”). Video consulting services were rarely, if ever, identified as a defined area for health policy and remained a fringe activity that was led by a small group of enthusiasts. Beacon sites existed however these developed *ad hoc* and seemingly with limited central support. With multiple platforms across primary and secondary care and reliance on bottom up, discretionary adoption, spread was limited.

As the pandemic hit, those leading digital innovation focused on engaging with Trusts and primary care providers to work with the small number of clinicians and their teams who had already successfully set up video consulting, run quality improvement programs to support interorganizational networks and peer-to-peer learning, and provide resources for providers and patients to support the use (and hence spread) of virtual consulting. With diverse video technologies and platforms already procured across the system—largely by individual providers, and with limited central input or guidance—development was *ad hoc*, locally driven and informed by existing infrastructure and capacity. In this sense, the response to the pandemic revealed significant gaps in national infrastructure and digital readiness, and dilemmas about how best to coordinate, organize and deliver health care at a time of crisis.

As the pandemic evolved, there was a continued emphasis on virtual consulting with support provided *via* an evolving quality improvement program. Consulting in Northern Ireland involved a mix of telephone and video, with in-person consulting as needed and in line with evolving guidance on physical distancing. A structured and systematic approach to virtual and video consulting remained the ambition however, while progress was clearly made, that ambition has yet to be achieved.

National level activity data is hard to come by, hence there is limited detailed evidence on how policy played out on the ground. Stakeholder interviews suggested wide variation in video consulting, with limited uptake in primary care. A press release from the British Medical Association Northern Ireland in September 2020 indicated that GPs across the country had “*carried out 14,000 video consultations*” (102) in the previous 6 months (i.e., since the pandemic started). It's not clear where this figure came from, or which platforms were used.

In sum, Northern Ireland was behind other nations in terms of digital health strategy and infrastructure. During the pandemic significant effort went into spreading virtual consulting, with video consulting one part of a blended approach. However, while quality improvement initiatives and shared learning about video consulting services helped, much of this effort was bottom up, led by frontline enthusiasts and often in spite of, rather than because of, national efforts.

### Cross National Comparison

**Figure 3:**
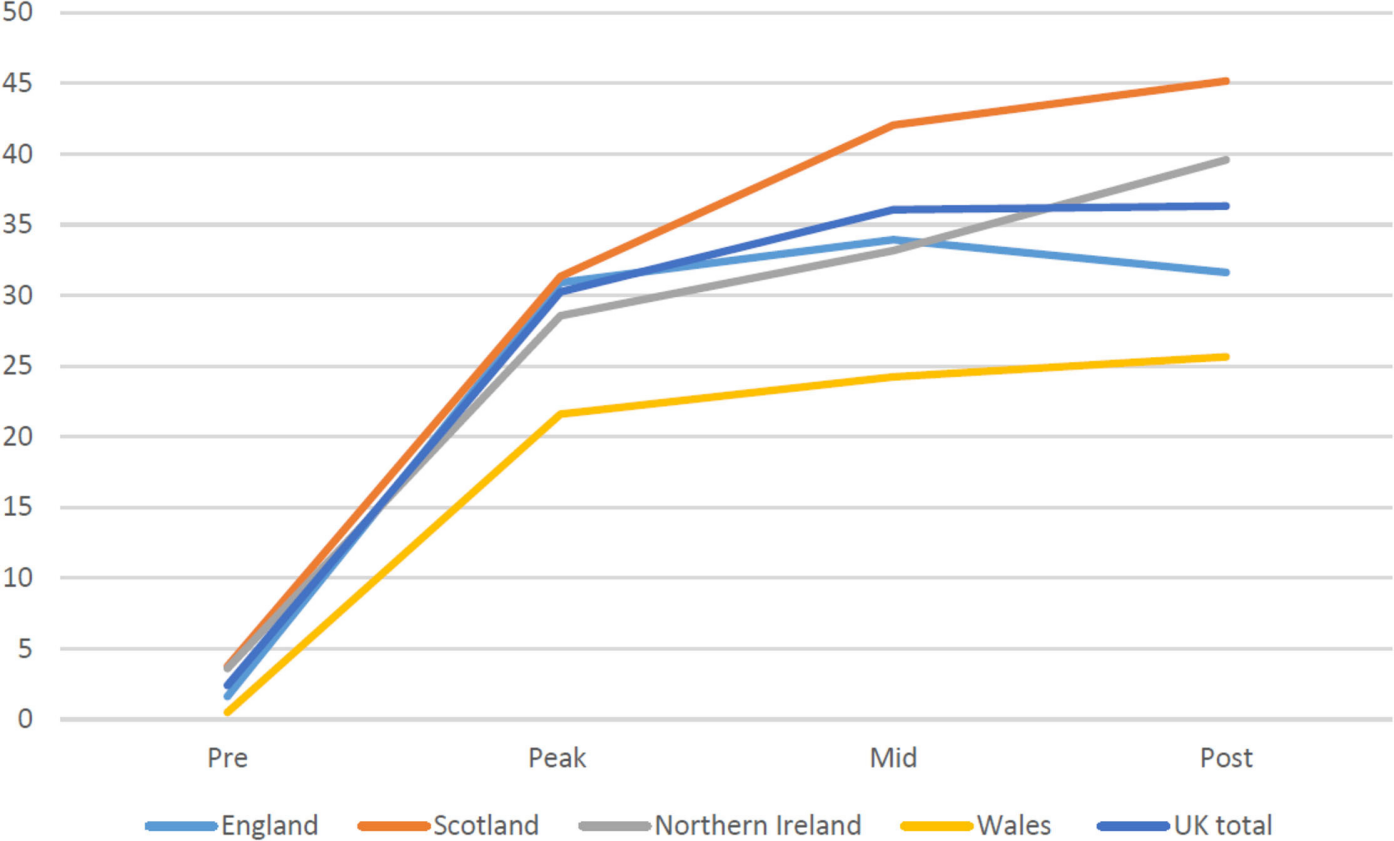
Reported proportion (%) of consultations carried out by video in each nation during the first 6 months of the pandemic. Data is taken from our national survey of NHS staff, conducted in September 2020, with pre-COVID before March, peak during March/April, mid during May/June and post in July/August.

National narratives on video consulting show that, even in the context of an unprecedented global emergency, establishing and sustaining video consultation services as business as usual is challenging. Despite calls from senior policymakers for “remote by default” services (103), analysis of interview, survey and activity data indicates variability in approach across the four nations, and in levels of spread and scale-up of video consulting (see Figure 3).

The following sections tease out similarities and differences in policy approach and areas of learning relating to spread, scale-up and sustainability of video consulting.

#### Infrastructure as the Foundation for Spread and Scale-Up

Across all four nations, the pandemic was situated as an opportunity (104) for rapid growth of video consulting services. Such spread and scale up was reliant (at least in part) on the extent of infrastructure, what Star referred to as “*what other things run on*” (105), including technical, material, operational and logistical arrangements. Infrastructure largely runs in the background and is visible only on breakdown (52, 105)—it requires foregrounding and active (usually long-term) planning as part of the policy process.

In Scotland, the Government's longstanding commitment to using technologies to achieve high-quality, accessible and equitable care and contribute to a low-carbon future meant that investment in digital infrastructure was well under way when the pandemic hit, providing strong foundations to rapidly scale-up its national video consulting programme. Scotland had foregrounded the on-going investment and work (105, 106) required to maintain and evolve digital infrastructure. For instance, while remote areas had limited or no broadband access (e.g., some outlying islands only had broadband outside the largest town for a few years), this was improving due to an on-going policy push for connectivity.

When the pandemic hit the pre-existing infrastructure in Scotland meant that the Near Me service could be immediately mandated across the country. This clearly paid off, with significant spread and scale up (Figures 2, 3). Elsewhere, while pockets of innovation existed (e.g., in specific regions) the national infrastructure required to rapidly spread video consulting in England, Wales and Northern Ireland was only partially in place. Challenges included rural broadband access (e.g., in parts of Northern Ireland), lack of bandwidth, outdated equipment, limited investment and staff training, and partial guidance and support. While England, Wales and Northern Ireland were considering or piloting Attend Anywhere, none had procured it (or any other platform). This was neatly summed up by one senior clinical decision maker in Wales, who told us, “*the fact that Attend Anywhere wasn't ready was the biggest challenge*”.

Recent and rapid investment helped. As a senior policymaker in Wales reflected: “*we had a lot of new…infrastructure, software as well as hardware, and so that helped because it could soak up the very sudden dial up in demand capacity that we needed*”. But on-going work and investment (pre- and in-pandemic) was needed. What this meant was that, while individual providers were clearly able to (in some cases, rapidly) develop video consulting services, the partial infrastructure and the on-going effort required to continually rework it as new resources and products came online, presented a major challenge to scale-up.

One aspect of infrastructure that all four nations focused on at the start of the pandemic was the removal of regulatory and administrative blocks. As one hospital doctor in Scotland put it, “*when COVID happened—the red tape seemed to vanish*”. This included removal of regulatory blocks to rapid procurement, the use of bespoke software for supporting video consultations (e.g., *via* procurement of Attend Anywhere in England and Wales), and relaxation of regulatory rules around information governance. As one senior policymaker in England told us in relation to data sharing:

“*nationally there's been information governance guidance that's gone out…that's been managed through the COVID notice which allows data sharing between [providers]…to make that information for direct care purposes available… traditionally, you know, going through data sharing agreements takes time…. Whereas actually this has been taken on centrally, some of that bureaucracy I suppose has been lightened in this crisis*”.

This focus on information governance was critical at the outset of the pandemic, shaping how and when video consulting was adopted and which platforms were used (e.g., some providers issued guidance to prevent use of Zoom early on in the pandemic due to concerns over privacy and security), and enabling spread.

#### Governance, Politics and Digital Technologies

Health system governance and politics shaped policy implementation. In England, guided by the market-oriented approach characteristic of the English NHS (Table 1), the crisis response to digital technology (104) was framed as an *opportunity* to innovate (Table 3), accelerate change and shift toward a “remote by default” model of health care (103). Freed from the fetters of heavy-handed state control in earlier “Big IT” projects [notably the UK's National Programme for Information Technology (NPfIT), a nationally-led program, characterized by centralized authority, that aimed to bring NHS “use of information technology into the 21st century”, but failed to deliver on a massive scale with costs to the UK taxpayer of over £13bn (107, 108)], the approach was to enable middle out and bottom-up change.

Previously the commissioner-provider split (in England since 1991, but resisted in Scotland, Wales and Northern Ireland—Table 1) has created practical hurdles for the introduction of new technologies (109). At the start of the pandemic two significant changes were quickly put in place to better manage the market: central procurement of a single platform (Attend Anywhere), and release of central guidance on procuring other platforms. This eased rapid set up and spread, particularly in secondary care:

“*we knew as a team the only way we were gonna get this off the ground quickly is to use Attend Anywhere, obviously it was a client procurement process, but…the end result was it has to be Attend Anywhere to make this happen because of all the learning through the pilot*” [Senior policymaker, England]

In Northern Ireland legacy agreements between suppliers and largely autonomous hospitals placed limits on the potential for national or regional coordination. As one policymaker told us: “*Because they [the five Trusts] cover a wide area and they have obviously a lot more autonomy, so they basically kind of wanted to do their own thing*”. Pockets of innovation enabled video consulting services to develop (notably in antenatal care). However, the challenges allied to developing digital infrastructure and of rapidly reorganizing services at a time of crisis, left few options for policymakers beyond continuing arrangements with multiple suppliers and using existing platforms.

In Scotland and Wales the aspiration was to develop national video consulting services. Rather than engaging multiple suppliers (as in England and Northern Ireland), the focus was on establishing and managing central provision. Prior policy and investment in Scotland, along with advanced digital infrastructure and the Near Me service, meant that the immediate pandemic response provided an opportunity to accelerate scale-up, extend successful models of good practice using principles of quality improvement (with central support, training and guidance, and system learning), and rapidly bring many providers to the same level as pre-pandemic exemplar sites.

In Wales, with one eye on the success of the Scottish service, the overarching crisis response was framed as an *opportunity* to become known as a national digital innovator—to build a national video consulting service and gain political and health system currency. The aspiration for a national service was well-received in government and TEC Cymru (see above). However, the reality on the ground was problematic: historic lack of infrastructure, limited resources, collective modes of decision-making and the work required to bring providers across sectors on board with the logistical, technical, material and cultural aspects of video consulting proved challenging. As one policymaker reflected, the pandemic brought opportunities to rethink this approach:

“*How do we move away from having two years to write a business case and that, you know, particularly IT—digital procurement—takes years for us to do and we'll buy some and it'll take seven years to implement. That's not always about procurement, that's about everybody agreeing what they want and how we're actually taking that forward. So, I think there are opportunities in this for us to rethink some of that*”.

Cross-national politics also played a role in shaping, at least peripherally, the approach to scaling up video consulting. Several interviewees described longstanding competition across Wales, Scotland and England (which typically fell out along party political lines) and spurred the vision for respective health systems to “lead the way” in technology-enabled care across the UK.

#### Making Policy Happen: Operational Crisis Management and the Spread of Video Consulting

It was the operational crisis management (32, 33) of senior civil servants and health service executives—focused on the implementation of evolving policy by frontline NHS staff—that shaped understanding of evolving policy, approach to implementation and what played out on the ground.

In Scotland and England this often (but not always) involved a pragmatic approach to both policy implementation and crisis management (33), with senior civil servants taking proposals to develop “remote by default” consulting and turning them into workable “real world” policies. Rather than relying on the kind of technocratic problem-solving that tends to be characterized as “policy” (e.g., with a series of linear steps involving problem definition, policy formulation, implementation and evaluation), these individuals and their teams focused on “making policy happen” at a time of crisis (33, 75). This process involved often senior and experienced staff in a process of sense-making (requiring judgements about set up and use of video consulting in the context of heightened ambiguity and uncertainty), decision-making and coordination (across multiple providers, suppliers, networks and political contexts; and involving legitimate explanation of decisions to NHS staff) and “learning-while-doing” (emphasizing adaption and bricolage in shaping video consulting services in the unfolding crisis response). Take the following example from a policymaker in England, supporting roll out of video consulting in secondary care:

“*It's very much carrot, not stick, mentality. And we've done some learning on quality improvement and things like that and…I'd say working with [head of directorate] it's been a completely different experience to any other role I've had in the NHS. It's just given the freedom and the space for us to kind of work in a different way and more of a, I guess you'd say more of a creative way*”.

Rather than being an explicit set of policy instruments or tools, this approach to “making policy happen” was grounded in practical rationality (33), coalition- and consensus-building (75) and use of process-oriented knowledge (75), while also acknowledging the unpredictability, uncertainty and complexity of the evolving system response to COVID-19 (64). Negotiating this terrain, while engaging and gaining support from NHS organizations and staff (e.g., regional leads, service managers) who were involved in, or could influence, the spread of video consulting, was critical to success. This was evident in the Scottish Government's enabling (rather than command and control) approach, with the TEC program creating the ethos and infrastructure within which professionals could be creative and locally adaptive; and with engagement of professional bodies (such as Royal Colleges) situated as critical in endorsing the TEC program's vision and guidance documents. Proactive communication between government, civil servants and professional bodies ensured that front-line clinicians believed that changes were professionally endorsed and led rather than imposed.

Evaluation and system learning was a key part of strategy in Scotland and England (and, to some extent, in Wales). We were particularly struck by the focus on region-by-region quality improvement in Scotland—and in secondary care in England—grounded in a system-wide approach that involved senior civil servants negotiating both political vision and frontline realities in ways that led to tangible and implementable actions (e.g., changes in procurement, technical guidance) that, in turn, supported the spread of video consulting.

In contrast, in Wales the operationalization of policy on video consulting was characterized by what policymakers and professionals described as centralized authority, a rigid approach to rollout and “*strongly embedded tribal interests from professional groups*”. While there were glimmers of system learning (“*everybody kind of came together*”), there was also tension across local and national interests that made it “*difficult to come to an all Wales consensus*”. In Northern Ireland, the focus remained largely on *virtual* (primarily phone) consulting, without an explicit policy on video consulting.

No matter the approach to making policy happen, cross-national exchange was critical in bolstering spread, enabling trusted relationships and sharing of expertise. As one program manager in Wales told us: “*We've certainly learned from NHS England; we've learnt those lessons [on confidentiality and governance challenges with video group clinics]*”. England, Wales and Northern Ireland all turned to the Scottish TEC team—before and during the pandemic—as a site of shared learning and, in England and Wales, a means of gaining additional capacity (e.g., *via* spare “waiting rooms” in the Scottish Near Me service). These links were not new, but were reinforced in the pandemic and proved critical in progressing rapid spread and scale-up.

#### Policy Diffusion vs. Bottom Up, Service-Led Adoption of Remote Services

Implementation of policy supporting spread of video consultations was a critical part of service change supporting the pandemic response (103, 110–112). However, the policy vision did not always match on-the-ground realities of health service provision. This was evident in primary care, where most consultations took place by telephone (37, 113–116).

GPs in our dataset repeatedly commented on how they reverted to use of the telephone first telling us, for instance, how “*I sometimes invite a patient to engage in a video consultation during a phone consultation, where I feel this would be helpful. I don't do it very often, as I am very used to the telephone consulting and find this adequate for around 90% of encounters*” (survey respondent), or how “*I mostly use telephone, sometimes use photos…*”. This was reflected in activity data, which showed only a small proportion of general practice consultations taking place *via* video (see e.g., Figure 2). Some GPs were uncomfortable with the video medium:

“*Having been quite pleased and quite excited by doing something new they then [after the first wave] became increasingly concerned that you hear lots of people saying, you know, ‘I didn't go into this business to be a call center doctor,’ ‘I like patient contact,’ ‘I feel unsafe’*” [Senior professional]

This “telephone first” approach was reflected across much of our dataset. In Scotland a high volume of GP practices introduced the Near Me service model, but use remained infrequent (23% of video appointment activity compared to 77% for hospital and other community services). A similar picture was evident in England where, despite rapid set up of 99% of practices at the start of the pandemic (set up being what one senior policymaker described as “*different from utilization, it's available in the practice*”), video consulting made up only a small proportion of general practice consultations. The focus was on digital triage, phone consulting and asynchronous e-consultations. As the same interviewee continued:

“*Our data shows they're increasing, I mean I think we're seeing about two and a half million a month being submitted…. When I'm talking about e-consultations I'm talking about this sort of route to access, asynchronous access to the practice: you get the information up front, and then you can kind of sort people to the right place*”.

This approach to remote consulting had received a significant policy push several years before the pandemic, with a drive for digital innovation in general practice and central funding to support it (78, 80, 117). Multiple suppliers came on board, with the technology typically service oriented, aligned with existing workflows and cheap to install. Comments (*n* = 575) from survey respondents across the four nations about choice of platform frequently referred to AccuRx, known to many before the pandemic (who were using it for texting patients), and considered readily available, easy to use and integrated with clinical systems. This resonates with the experience in Wales where the national video consulting service, using Attend Anywhere, was perceived by some to be aligned with secondary care workflows. GPs often reverted to AccuRx, “*which offered some really GP specific functionality, and there was…a misunderstanding about what was it GPs wanted or needed from video consulting software; so GPs wanted something to integrate with their practice systems*” [senior policymaker]. This made sense given that AccuRx was sold direct to practices (and was freely available during the first few weeks of the pandemic) as a general work support tool: as one industry representative told us, the focus is on “*trying to show primary care that it is possible to have software that works and is intuitive and its reliable*”.

Through this lens, policy implementation during the pandemic was unsuccessful. Rather, primary care provides a good example of bottom up, service-led adoption of a technology that was perceived as a good “fit” with existing workflows and clinical systems.

#### Longer Term Sustainability of Video Consulting

Moving beyond the initial crisis response, the ways in which the four nations made sense of the evolving pandemic and use of digital technology shifted to one of increasing exposure, highlighting “*the significance, actions and issues of people, social groups, systems, organizations and infrastructure that have previously gone unnoticed”* [(104), p. 5]. The focus across the four nations (albeit to varying degrees) was on three practical and moral questions about the organization and delivery of health services issues.

First, was increasing concern about digital exclusion of some patients and families, including potential longer term consequences:

“*We need to do more for people who don't have access to broadband or can't afford a laptop. … I don't believe that [remote] should be the default. We've come a long way in healthcare, we don't want to ruin it now*” [Service Manager, Scotland]

This raised moral questions for policymakers about how to balance the desire for digital transformation of health services with the need to ensure patients weren't excluded from services, or disadvantaged when they did so remotely [see (57) for examples].

Second was the level of uncertainty about the future organization and delivery of services. Three system issues were pertinent. Firstly confusion, especially in England and Wales, about whether the NHS is a “remote by default” service, with interviewees often uncomfortable with the push for everyone to access care remotely. Secondly the impact on the health care workforce, with some clinicians describing the use of video consulting (and the speed and scale of the switch) as demotivating, devaluing and challenging their sense of professional purpose and identity. Thirdly, how services would be redesigned as the pandemic evolved and what this meant for patients. As one program manager in Wales reflected in relation to group video consulting:

“…* we don't see it as a response to COVID; we see it as a response to COVID recovery planning, but also as a sustainable business-as-usual approach as part of that outpatient service delivery toolkit*”

Our dataset was peppered with similar examples situating video consulting as an integral and on-going part of the NHS offer (rather than a temporary response to the pandemic). This aligned with the renewed interest of politicians who were now: “*really excited about the speed with which digital transformation has changed…and at that policy level it's really helped to help people to grasp how digital can help drive change”* [Senior policymaker]. This interest was often couched in deterministic terms, seeing technology as a “quick fix” to problems of service delivery and redesign and failing to acknowledge the social-technical work involved in spreading and scaling up digital innovation. Work was underway to manage expectations:

“*I spend quite a lot of my time trying to talk people down a little bit… For some things we maybe could aim for five months, but there are also some things that are just inherently complex where we've got a lot of dismantling…and rebuilding to do”* [Senior policymaker, Wales].

Finally, there was significant concern about a return to pre-pandemic levels of governance and regulation. As one senior decision maker put it, “*there's this tension between how much do we maintain the lightweight rapid governance that we had versus how much do we bring back a degree of stability”*. Interviewees across all four nations repeatedly told us that this light touch regulatory approach was critical for spread and scale-up.

## Discussion

### Summary of Key Findings

In this paper we have focused deliberately on policy informing the spread and scale-up of video consulting services across the four UK nations, with the COVID-19 pandemic a burning platform for change. Drawing on data from three studies we have shown the following. First, an interpretive approach to policy analysis combined with theory on crisis management has allowed us to surface the varied national approaches to developing and enacting policy during the pandemic, the challenges faced by national decision makers in negotiating complex systems at a time of crisis, and the varied national policy-level influences aiding progress toward rapidly scaling up video consultation services. Second, following from this, we have shown how different approaches to understanding, negotiating and enacting policy during a time of crisis, variably shape spread and scale-up. Through the combined lens of policy implementation and crisis management, it is clear that those who are able to work flexibly and adaptively in the midst of an evolving crisis appear to be more effective in enabling the spread of video consulting services. Such work involves facilitating the capacity and articulation work needed to enable an iterative approach to implementation; involving multiple actors across the system to work together to solve emergent problems; engaging with processes and actions over time [rather than discrete decisions administered at a particular moment in time (72)]; and continually reviewing, monitoring and evaluating progress as part of a wider approach to quality improvement and system-level learning.

Third, we have shown how digital infrastructure (and on-going investment in, and adaptation of, that infrastructure) is foundational—without it, national-level scale-up is nigh on impossible even during a crisis. Investment in digital infrastructure in Scotland in particular is evidence of this—not only was Scotland uniquely well placed to expand its video consultation services at pace and scale when the pandemic hit (leading to a dramatic increase in the number of services adopting video and in consultations conducted), but the level of *infrastructuring* involved—i.e., the “*continuous collaborative and inherently political process*” [(118), p. 205] supporting iterative design and development of digital infrastructure—enabled continuous, steady and sustainable growth in ways that was appropriate to different sectors, organizations and needs.

Fourth, in some settings (often where a more technocratic policy approach was in play) we found a contrast between “work as imagined” by policymakers and “work as done” by frontline practitioners. This was evident in primary care where, in spite of significant policy enthusiasm for video consulting in England, Wales and Scotland, many clinicians reverted to using existing ways of remote consulting (e.g., telephone consultations), that aligned closely with clinical workflows and practices.

Finally, while there has been significant (if varied) spread of video consulting across the UK during the pandemic, findings indicate that sustainability of these services and potential for further spread will only be feasible if questions about the future shape of service delivery and resolution of digital inequalities are addressed.

### Strengths and Limitations

Our data is drawn from three studies that, together, provided a rare opportunity for cross-national analysis, enabled significant insights on evolving policy relating to video consulting, and shone a light on issues of policy implementation that have largely been ignored in literature on telehealth and video consulting to date.

Our dataset brought together national survey and interview data, with analysis of documents and activity data. While every effort was made to identify a diverse group of stakeholders in each of the four nations, the level of engagement from senior politicians at a time of crisis was limited. Those we did interview were able to provide a policy narrative on the spread and scale-up of video consulting. In this sense, we were able to access the national-level perspectives needed in each of the four nations and compare across these. UK-wide activity data was harder to come by. There is no readily available central dataset on consulting activity across the UK; and in England, Wales and Northern Ireland there is no readily available national dataset of consulting activity. In England we were able to access Attend Anywhere data for secondary care *via* NHSE/I (in line with confidentiality and data sharing agreements), but no data on use of other platforms, or in primary care. In Scotland, we were able to access national level data on the use of the Near Me service (using Attend Anywhere) but, again, no data on the use of other platforms. This means that, while the activity data we have provides a helpful snapshot, it falls short of providing a full picture of activity and, as different countries use different approaches and criteria for monitoring activity, cannot be used to draw direct comparisons. We only have reported data for Wales and Northern Ireland. Finally, our studies were primarily qualitative, focusing on the experiences and perspectives of those involved in setting up and running video consultation services at multiple levels. We are therefore unable to make a causal link between specific policy initiatives on the one hand and the spread of video consulting on the other.

### What Our Findings Add to Existing Literature

Our findings add to the literature on video consulting, which has tended to focus on specific clinical setting or condition, pay limited attention to policy initiatives and/or processes, and look at implementation *within* services, rather than knowledge sharing and other learning needed to achieve spread and scale-up of video consulting *across* settings (26). Comparison across countries is helpful here. Findings indicate that there was significant and rapid effort at system level in Scotland, England and Wales (less so Northern Ireland) to give space and impetus to scaling-up video consulting services in the midst of the pandemic, both by *national level* decision makers (e.g., civil servants) and *street level* bureaucrats (e.g., health service executives). The legacy of Scottish policy supporting video consulting, combined with the explicit focus on developing a national program, clearly enabled a rapid and coherent response in the midst of crisis. That the other three nations turned to Scotland for advice and support is telling in terms of the on-going need for system-level learning and exchange.

Disruptive technological innovation has been shown to be complex, uncertain, challenging and risky (74, 119, 120), with success not just about new technologies but also how we make them work and whether health service infrastructure can accommodate them at speed and scale (52, 121). This kind of infrastructure takes time and effort to develop and is achieved incrementally (105) with, for instance, new devices and platforms requiring reorientation and reworking of existing infrastructure over time (52, 118). For video consulting this includes hardware and software, as well as the language of clinical applications, a human-computer interface, people who interact with it (including developers, support staff, staff and patients), internal organizational features (e.g., environment, policies, procedures), the scaffold for a learning health system, external rules and regulations, and the measures and metrics used to monitor it (106, 122). This resonates with our findings: on-going work and investment in infrastructure in Scotland enabled the vision and foundations to support adoption to be in place ahead of the pandemic and on which rapid scale-up of the national video consulting program was possible. Elsewhere, limited investment in infrastructure and lack of infrastructuring pre-pandemic placed limits on spread and scale up in-pandemic.

The crisis context gave a critical boost to the implementation of policy on spread and scale up of video consulting. To our knowledge, this focus on crisis and the intersection with implementation of video consulting is new. A small number of pre-pandemic studies explored the technological, contextual and practical challenges to be overcome pre-pandemic for video consulting to be more widely used. One multi-level, qualitative study in the English NHS, undertaken by our team, examined the development, implementation and use of video consultation services (1, 51), focusing on national-level policy, organizational-level implementation, and micro-level use of video consultations within patient-clinician consultations. A key finding was the distinct mismatch between the policy narrative of transformation and efficiency (to be achieved through technological innovation) and the reality of services *not* being transformed by the available technology (which may be experienced as unfit for purpose). Findings from our research suggest that this policy narrative, and the mismatch with frontline experiences, has continued during the pandemic: what decision makers viewed as an *opportunity* to scale up video consulting appeared to be at odds with what many in primary care viewed as *disruption* in terms of the work involved in “*rapidly shifting existing organizational practices to new digital spaces*” [(104), p. 4]. This builds on earlier work on technological innovation in health care and the challenges of routinizing new technologies in everyday practice [e.g., (48, 74, 123)].

The approach to policy implementation and crisis management in each of the four nations was key in rapidly spreading and scaling up video consulting. To date, research has tended to focus on the political and institutional context of policy making at a time of crisis or on strategic crisis management [see e.g., (124, 125)]. Our research adds to this growing body of work, foregrounding *approaches to crisis management* that are informed by complex systems thinking and notions of unpredictability, radical uncertainty, non-linearity, and adaptability (60, 61, 63, 126); and effective *implementation of policy* as involving pragmatic and iterative cycles of sense-making, meaning making and learning-while-doing (33, 34, 75). In short, an approach to implementing policy on video consulting that is grounded in pragmatism and practical rationality appears more likely to facilitate spread—particularly at a time of crisis—than one grounded in technocracy and technological determinism (33).

### Conclusions and Recommendations

Pre-pandemic video consulting was a marginal activity (1, 58). This changed with COVID-19. The combination of a highly infectious disease and requirements for physical distancing, with increased funding, relaxed regulation, engaged suppliers and an approach to “making policy happen” (33, 64), enabled spread of video consulting at a pace and scale that was previously unimaginable. This shift was logistical, as well as technical and cultural, and required significant policy input. Further spread may well have been possible, however, digital infrastructure was only partially in place hampering speed and scale of progress. As the acute phase of the pandemic passes, senior decision makers would do well to: (a) advance the infrastructural building blocks that are now in place to support video consulting services, (b) recognize and accommodate the level of infrastructuring (118) required to sustain and extend scale-up going forward, and (c) take an active interest in the ways in which the policy process—particularly implementation—can be further strengthened and supported. This is critical if the UK NHS is to be ready in the face of further unexpected and rapidly evolving crises that require foundations for action to be in place, and rapid application of plans and skills. As Boin so neatly puts it, “*the leadership challenge is to have good plans and professional responders in place*” [(32), p. 8/9].

Consideration of the longer term sustainability of video consulting services will be crucial given the policy vision for video consultations as replacing or supplementing a significant proportion of in person care (79, 127, 128). The jury is still out with regard to if and how nationally coordinated (as in Scotland and Wales) and locally devolved (as in much of England and Northern Ireland) video consulting services are best placed to enable continued scale-up and the extent of sustainability offered by different approaches in the longer term. There is much to learn across the four nations: research is needed that focuses, not only on design, development, procurement and regulation of different kinds of video consulting services (e.g., national/local, one/many suppliers), but also on the cross-national learning that can support effective policy implementation, crisis management and spread and scale-up.

Scale up of video consulting during the pandemic has exposed the lack of attention previously given to those with limited access to services and digital resources [in terms of “*magnified high levels of inequality*”, [(104), p. 6]. Some work is already underway to redress this, but policymakers and researchers need to do more to improve uptake and ongoing use of video consultation services for marginalized and/or underserved groups (129). Without this, further scale-up and longer term sustainability of video consulting services is unrealistic and the potential to respond quickly and appropriately in the face of similar crises limited.

## Data Availability Statement

The raw data supporting the conclusions of this article will be made available by the authors, without undue reservation.

## Ethics Statement

The studies involving human participants were reviewed and approved by NHS East Midlands Leicester Central Research Ethics Committee granted research ethics approval for the study (REC ref 20/EM0128; IRAS ID: 283196 and subsequent amendments). The patients/participants provided their written informed consent to participate in this study.

## Author Contributions

SS leads a work package on the Remote by Default study and is Chief Investigator on the Health Foundation Video Consultation study. She provided theoretical expertise, led senior stakeholder interviews (with support from AR, RR, and TG) and documentary analysis, and drafted the paper. TG is Chief Investigator of the Remote by Default study and joint Chief Investigator (with JW) of the Scottish telehealth evaluation study. RR leads a work package on Remote by Default. CP is lead for group video consulting on the Health Foundation study. GH, LM, and JW led on interviews across the four nations. GW led on survey design and reporting, AR on analysis of the Welsh case study, and JM on patient experiences of video consulting. TG, RR, and LM contributed clinical and some social science and technology expertise. SS, GH, JW, CP, AR, JM, GW, and SF contributed social science and technology expertise. All authors contributed to the empirical studies and selected and supplied empirical data that illustrated and shaped the analysis. All authors have read and approved the final manuscript.

## Funding

This research was funded from the following sources: Scottish Government (Technology Enabled Care program), National Institute for Health Research (BRC-1215-20008), UK Research and Innovation *via* ESRC (ES/V010069/1), Wellcome Trust (WT104830MA), and the Health Foundation, an independent charity committed to bringing about better healthcare for people in the UK (2133488).

## Conflict of Interest

The authors declare that the research was conducted in the absence of any commercial or financial relationships that could be construed as a potential conflict of interest.

## Publisher's Note

All claims expressed in this article are solely those of the authors and do not necessarily represent those of their affiliated organizations, or those of the publisher, the editors and the reviewers. Any product that may be evaluated in this article, or claim that may be made by its manufacturer, is not guaranteed or endorsed by the publisher.
